# Head-to-head comparison of multiple cardiovascular magnetic resonance techniques for the detection and quantification of intramyocardial haemorrhage in patients with ST-elevation myocardial infarction

**DOI:** 10.1007/s00330-020-07254-1

**Published:** 2020-09-14

**Authors:** Anna Giulia Pavon, Georgios Georgiopoulos, Gabriella Vincenti, Olivier Muller, Pierre Monney, Gregoire Berchier, Chiara Cirillo, Eric Eeckhout, Juerg Schwitter, Pier Giorgio Masci

**Affiliations:** 1 Centre of Cardiac Magnetic Resonance – Lausanne University Hospital, Lausanne, Switzerland; 2 Cardiology Division, Heart & Vessels Department, Lausanne University Hospital, Lausanne, Switzerland; 3 Cardio-Thoracic-Vascular Department, San Raffaele Scientific Institute, Milan, Italy; 4 School of Biomedical Engineering and Imaging Sciences, King’s College London, St Thomas’ Hospital, Westminster Bridge Road, London, SE1 7EH UK; 5 Faculty Biology and Medicine, Lausanne University, Lausanne, Switzerland; 6 Radiology Department, Lausanne University Hospital, Lausanne, Switzerland

**Keywords:** Myocardial infarction, Magnetic resonance imaging, Myocardial ischemic reperfusion injury

## Abstract

**Objectives:**

T2*-weighted (T2*w) is deemed as a reference standard for post-infarction intramyocardial haemorrhage (IMH). However, high proportion of T2* images is affected by off-resonance artefacts hampering image interpretation. Diagnostic accuracy and precision of alternative techniques for IMH diagnosis and quantification have been seldomly investigated.

**Methods and results:**

Between April 2016 and May 2017, 50 ST-segment elevation myocardial infarction patients (66% male, 57 ± 17 years) and 15 healthy controls (60% male, 58 ± 13) were consecutively enrolled. Subjects underwent head-to-head comparison of single mid-infarct slice acquired on black-blood T2-weighted short-TI-inversion recovery (T2w-STIR), bright-blood T2prep-steady-state-free precession (T2prep-SSFP), and T2/T1 maps for IMH diagnosis and quantification against T2*w. All images were graded for quality (grade 1: very poor; grade 4: excellent) and diagnostic confidence (Likert scale, 1: very unsure and 5: highly confident). Reduced relaxation time/hypointense region (hypocore) embedded in infarct-related oedema on T2 map, T1 map, and T2w-STIR had the best overall diagnostic accuracy (per-subject: 91%, 86%, and 86%, respectively; per segment: 95%, 93%, and 93%, respectively). By mixed-effects analysis, image quality, and diagnostic confidence were higher for T2 map and T1 maps than T2*w (*p* < 0.05 for both scores). For IMH quantification, hypocore on T2 map and T1 map strongly correlated (Spearman’s *r* > 0.7, *p* < 0.001 for both) with IMH extent on T2*w and presented an overall excellent agreement on Bland-Altman analysis. By linear mixed model analysis, absolute hypocore size did not differ among T1-, T2 map, and T2*w. T2/T1 maps had the best intra- and inter-observer reproducibility among CMR techniques.

**Conclusion:**

Hypocore on T2/T1 map is the best alternative technique to T2*w for diagnosing and quantifying IMH in post-STEMI patients.

**Key Point:**

*• Mapping techniques are the best alternatives for diagnosing post-infarction intramyocardial haemorrhage.*

*• Mapping techniques are valuable tools for imaging intramyocardial haemorrhage.*

**Electronic supplementary material:**

The online version of this article (10.1007/s00330-020-07254-1) contains supplementary material, which is available to authorized users.

## Introduction

Cardiovascular magnetic resonance (CMR) represents a valuable non-invasive modality for studying the ischemia/reperfusion (I/R) myocardial injury in patients with ST-segment elevation myocardial infarction (STEMI) [1]. Intramyocardial haemorrhage (IMH) is a marker of severe I/R damage being associated with microvessel wall destruction and interstitial erythrocyte extravasation [2]. In experimental and clinical studies, IMH is related to unfavourable clinical outcomes [3–14]. Thus, non-invasive detection and quantification of IMH by CMR may play a key role in the risk stratification of STEMI patients as well as in the development of an imaging-based biomarker for testing treatments aiming to minimise I/R damage and improve patients’ prognosis [15, 16]. To date, T2* mapping is claimed to be the ‘reference standard’ for post-infarction IMH detection and quantification [17, 18]. Multi-echo T2* imaging represents the comparator against which other imaging techniques are evaluated. However, the inclusion of IMH assessment in clinical studies has been hindered due to the fact that multi-echo T2* imaging is prone to off-resonance artefacts resulting in a relevant proportion of patients with uninterpretable images [19]**.** Moreover, multi-echo T2*w imaging does not allow for the concomitant detection and quantification of infarct-related oedema, which provides relevant complementary information in STEMI [20]. Black-blood T2-weighted (T2w) short-TI-inversion recovery (STIR) and bright-blood T2prep steady-state-free precession (SSFP) as well as T1 mapping (T1 map) and T2 mapping (T2 map) have been used in previous studies for the visualisation and quantification of ischemia-related oedema and IMH [3–14]. With respect to IMH identification and quantification, these studies were limited by the lack of a properly defined reference standard, spectrum bias due to the absence of a healthy control group and the absence of a direct comparison among the diverse techniques. They also seldomly reported the precision of the techniques for IMH quantification, essential information for sample size calculation when planning randomised controlled trials.

Based on these premises, we studied a cohort of STEMI patients and healthy control using T2w-STIR and T2prep-SSFP as well as T2/T1 maps and multi-echo T2*w imaging. We set T2*w imaging as the reference standard, and we hypothesised that, when compared with T2*w, CMR alternative techniques may (1) be associated with improved image quality, diagnostic confidence, and non-inferior diagnostic accuracy for IMH detection; (2) provide reliable surrogate estimates of IMH (for mapping techniques); and (3) offer better intra- and inter-observer reproducibility.

## Materials and methods

### Study population

Between April 2016 and May 2017, 61 STEMI patients were consecutively evaluated for study inclusion at the Lausanne University Hospital. Inclusion and exclusion criteria are provided in the Supplementary Material. Fifteen age- and gender-matched subjects, in whom ischemia and structural heart disease were excluded by comprehensive CMR at the same institution, were recruited as healthy controls. Institutional review board approval (institutional review approval: PB_2016-02583 – 06/10) and subject’s informed consent was obtained. None of the subjects participated in previous studies. The study was not registered to the Clinical Trial Registry or equivalent registries.

### Cardiovascular magnetic resonance protocol

All subjects underwent CMR at 1.5-T scanner (Aera-Magneton, Siemens Healthcare) after a mean period of 3 ± 2 days from index primary percutaneous coronary intervention (PCI) as described in detail in the Supplementary Material. Short-axis cine images were readily evaluated during the CMR scan by an experienced operator (P.G.M), and the short-axis slice showing the most extensive wall motion abnormalities was selected as the target slice (Supplemental Material). In target-slice position, the following sequences were acquired: (1) T2w-STIR; (2) T2prep-SSFP; (3) T1 map; (4) T2 map; and (5) multi-echo T2*. Sequence parameters are reported in Supplementary Table 1.

## Image analysis

### Definitional gold standard for IMH in the target slice

A side-by-side visual comparison between LGE target slice and multi-echo T2* was used to localise the infarct region on the corresponding T2* images. Intramyocardial haemorrhage was defined as sub-endocardial to mid-wall hypointense signal within the infarct region on the multi-echo T2*w image with the longest echo time (i.e. 16.22 ms) (T2*w) [10]. Hypointense signal on T2*w image outside the infarct region or confined to the epicardial layer of infarct was deemed as off-resonance artefact. When present, IMH was allocated to a specific segment based on LV segmentation of the American Heart Association [20]. IMH was quantified as hypointense signal within the infarcted myocardium showing signal intensity < 2 standard-deviations of the mean signal intensity of the remote myocardium [9, 10].

### Target slice image analysis

**Fig. 1:**
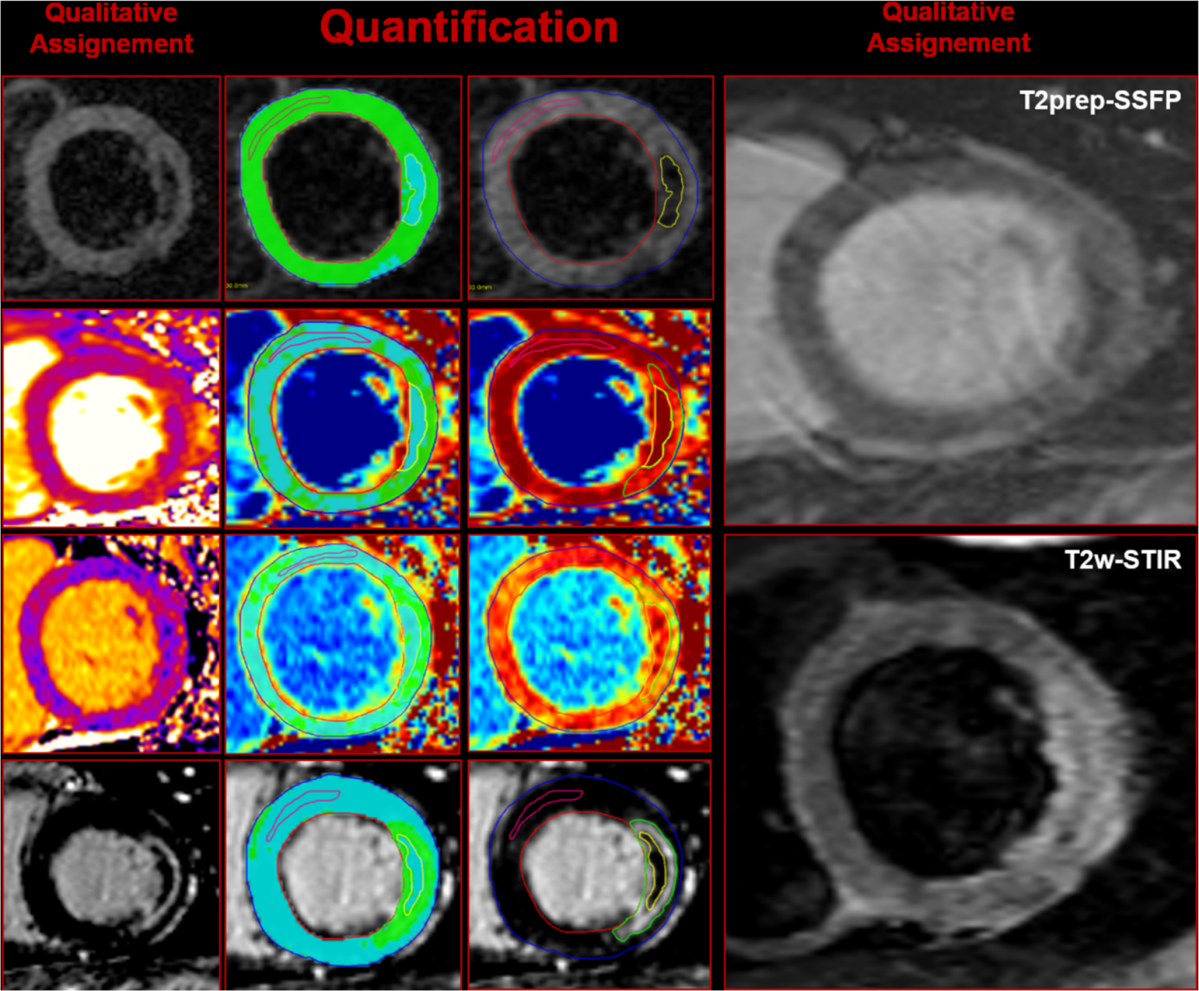
Qualitative and quantitative assessments of the target slices. For T2*w, T2/T1 maps, and LGE, the analysis started with the qualitative evaluation (first column) to continue then with the quantitative assessment (2nd and 3rd columns). For T2w-STIR and T2prep-SSFP, only the qualitative assessment is carried out (4th column). LGE and MVO quantifications were not included in the comparative analysis with T2*w for IMH. *Abbreviations*: T2*w, T2*-weighted; T2 map, T2 mapping; T1 map, T1 mapping; IMH, intramyocardial haemorrhage; T2prep-SSFP, T2 preparation steady-state-free precession; T2w-STIR, T2-weighted short-TI inversion recovery; MVO, microvascular obstruction; LGE, late gadolinium enhancement

An experienced CMR technician (G.B) blinded to patients’ clinical history and CMR results anonymised patients’ and healthy controls’ DICOM target slices and uploaded them in a dedicated workstation with a random order using vendors’ independent software (GTVolume, Version 2.2.1; GyroTools). The same operator (PGM) analysed all studies. Target-slice assessment started with a 4-grade image quality score: (1) extensive artefacts not allowing image interpretation; (2) moderate artefacts not hampering image interpretation; (3) mild artefacts with overall good image quality; and (4) absence of artefacts with excellent image quality. Only target slices graded ≥ 2 were then analysed. The operator’s confidence for binary assignment of IMH was graded according to 5-point Likert scale (grade 1: 10% confidence, very unsure, to grade 5: 90% confidence, highly confident) [21]. The same operator re-evaluated all target slices 1 month apart (i.e. 50 patients and 15 healthy controls) for intra-observer reproducibility. Another experienced operator (A.G.P.) in CMR analysed blindly all target slices for inter-observer reproducibility. Target slices of 16 patients showing IMH on T2*w images and concomitant hypocore on T2 map and T1 map were re-analysed for testing intra- and inter-observer reproducibility of quantitative data. Hypocore was defined as the hypointense region embedded in the infarcted myocardium. An example of both qualitative and quantitative analyses of the target slice is shown in Fig. 1. Detailed qualitative and quantitative target-slice analyses are reported in the Supplemental Material. LV volumes, mass, and ejection fraction were calculated as previously reported [3–22]. Infarct-related oedema, hypocore, infarct size, and MVO were calculated for the LV using the same post-processing algorithm described for the target slice and the results are reported in Supplementary Table 2.

### Statistical analysis

Differences in qualitative features across CMR techniques were evaluated by multilevel mixed-effects ordered logistic regression. CMR technique diagnostic accuracy was measured on a per-subject and a per-segment basis using the T2*w as the reference standard (“true diagnosis”). We estimated sensitivity, specificity, false and negative rates, and positive (PPV) and negative (NPV) values [23]. Receiver operating characteristic (ROC) analysis was used to compute the area under the curve (AUC) which provided the discriminative ability of CMR sequences against the reference standard (IMH presence on T2*w). We analysed repeated quantitative measurements of hypocore size by implementing generalised estimating equations (GEE) with unstructured variance-covariance matrix. The agreement between IMH on T2*w and hypocore on T2/T1maps was assessed by (i) correlation coefficients; (ii) Bland-Altman analysis. Intra- and inter-observer (precision) reproducibility of T2*w and other diagnostic techniques for IMH diagnosis were assessed by Cohen’s kappa and intra-class correlation coefficients (ICCs) for the same and different operators. Statistical analysis was performed with STATA package, version 11.1 (StataCorp). All tests were 2-tailed, and *p* < 0.05 was considered statistically significant. Detailed statistical methods are provided in the Supplementary File.

## Results

### Study population

**Fig. 2:**
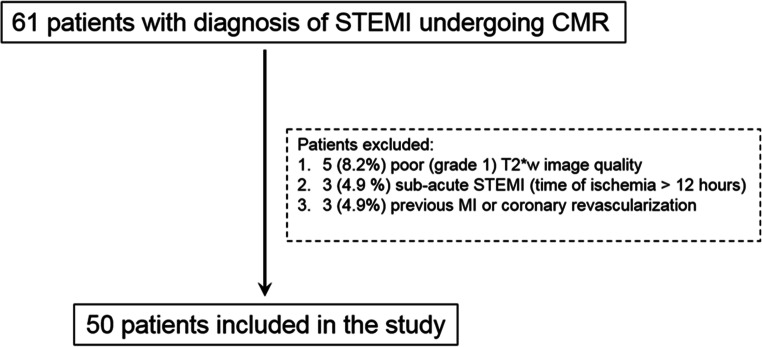
Flow chart of the study

Among 61 STEMI patients evaluated for inclusion to the study, 5 (8%) and 6 (20%) patients were excluded because of poor T2*w image quality (grade 1) and previous MI/coronary revascularisation, respectively (Fig. 2)**.** All study patients were treated according to the current guidelines for STEMI [24]. Baseline clinical characteristics of STEMI patients are summarised in Table 1. Healthy controls had similar age (57 ± 17 vs 58 ± 13 years, *p* = 0.706) and gender distribution (male 66% vs 60%, *p* = 0.761) compared with STEMI patients (Supplementary Table 3). Overall, the study cohort comprised 65 subjects (50 STEMI patients and 15 healthy controls). The target slice was located in basal/mid in 62 and in apical position in 3 subjects, yielding a total of 384 segments (average number of segments per enrolled subject was 5.9).

**Table 1:** Characteristics of STEMI patients

Variable	Patients (*n* = 50)
Age, years	58 ± 13
Male gender, *n* (%)	30 (60)
Family history for CAD, *n* (%)	7 (14)
Diabetes, *n* (%)	3 (6)
Hypertension, *n* (%)	26 (52)
Hypercholesterolemia, *n* (%)	27 (54)
Smoking, *n* (%)	22 (44)
Prodromal angina, *n* (%)	10 (20)
Systolic BP (mmHg)	125 ± 27
Diastolic BP (mmHg)	69 ± 15
Heart rate (bpm)	75 ± 16
*Peak CPK (units/L)	1376 (534–2555)
*Peak CPK-MB (units/L)	200 (98–369)
Time-to-PCI (min)	182 ± 56
RPP before PCI (mmHg × bpm)	9375 ± 2680
PCI-to-CMR interval, days	3 ± 2
Infarct-related artery, *n* (%)	
LAD	20 (40%)
Proximal	16 (80%)
Mid	2 (10%)
Distal	2 (10%)
RCA	14 (28%)
Proximal	9 (64%)
Mid	3 (21%)
Distal	2 (14%)
LCX	16 (32%)
Proximal	8 (50%)
Distal	8 (50%)
TIMI flow grade pre-PCI, *n* (%)	
0, 1	26 (52%)
2, 3	24 (48%)
TIMI flow grade post-PCI, *n* (%)	
0, 1	13 (26%)
2, 3	37 (74%)
Rentrop flow grade, *n* (%)	
0, 1	47 (94%)
2, 3	3 (6%)
Right-dominant coronary circulation	45 (90%)
Left-dominant coronary circulation	5 (10%)
Non-IRA critical stenosis, *n* (%)	
0 vessels	18 (36%)
1 vessel	14 (28%)
2 vessels	18 (36%)
Medication at discharge	
ACEi or ARBs, *n* (%)	45 (90%)
Beta-blockers, *n* (%)	35 (70%)
Statins, *n* (%)	40 (80%)
Diuretics, *n* (%)	7 (14%)

*ACEi*, angiotensin-converting enzyme inhibitor; *ARB*, angiotensin receptor blocker; *BP*, blood pressure; *CAD*, coronary artery disease; *CMR*, cardiovascular magnetic resonance; *CPK*, creatine phosphokinase; *IRA*, infarct-related artery; *LAD*, left anterior descending artery; *LCX*, left circumflex; *MB*, myocardial band; *PCI*, percutaneous coronary intervention; *RCA*, right coronary artery; *RPP*, rate pressure product; *TIMI*, thrombolysis in myocardial infarction

*median and interquartile range provided

### Qualitative target-slice results

T2*w image quality was good (grade 3) to excellent (grade 4) in 21 (32%) and 43 (66%) subjects, respectively (quality score: 3.6 ± 0.5). The diagnostic confidence for T2*w was good (grade 4) to excellent (grade 5) in 17 (26%) and 44 (68%) subjects, respectively (mean score: 4.6 ± 0.7). All T2 maps and T1 maps were judged good or excellent (grade 3 or 4), whereas T2-STIR and T2prep-SSFP were scored very poor quality (grade 1) in 2 (4%) cases. Accordingly, all parametric images (*n* = 65) and 63 images of T2-STIR and T2prep-SSFP were included in the analysis. The mean image quality and diagnostic confidence based on Likert’s scale are reported in Table 2. By mixed-effects ordinal logistic regression, image quality was better for T1 map (*p* = 0.047) and T2 map (*p* = 0.001). In terms of diagnostic confidence, T2 map was not inferior to T2*w (*p* = 0.676). In contrast, all other imaging techniques had lower diagnostic confidence than T2*w (*p* < 0.001) (Table 2).

**Table 2:** Per-subject and per-segment diagnostic accuracy of the diverse techniques for IMH diagnosis

Technique	Image quality	Diagnostic confidence	Sensitivity (95%CI)	Specificity (95%CI)	False-positive rate (95%CI)	False-negative rate (95%CI)	NPV (95%CI)	PPV (95%CI)	AUC (95%CI)	Diagnostic accuracy (95%CI)
Per-subject										
T2w-STIR	3.6 ± 0.7	4.1 ± 0.9	^†^100 (100–100)	^†^84.8 (80.5–88.4)	^†^15.2 (11.6–19.5)	^†^0 (0–0)	^†^100 (100–100)	^†^72.7 (65.4–78.6)	0.92 (0.90–0.92)	89.1 (85.9–91.9)
T2prep-SSFP	3.5 ± 0.7	4.0 ± 1.0	^†^100 (100–100)	^†^76.1 (71.7–80.4)	^†^23.9 (19.6–28.3)	^†^0 (0–0)	^†^100 (100–100)	^†^62.2 (56.3–67.7)	^†^0.88 (0.86–0.90)	^†^83.1 (79.7–85.9)
T2 map	*4.0 ± 0.1	*4.6 ± 0.6	95 (91.7–100)	89.4 (846.1–92.5)	10.6 (7.45–14)	8.33 (5–12.5)	97.7 (95.8–100)	79 (72.7–84.6)	0.92 (0.90–0.94)	90.8 (87.7–93.9)
T1 map	*3.9 ± 0.4	*4.0 ± 0.9	^†^84.6 (78.6–90)	^†^87 (83.7–90.5)	^†^15.4 (10–21.4)	^†^13 (9.5–16.3)	^†^93.1 (90.6–95.5)	^†^72.7 (65.4–79)	^†^0.86 (0.82–0.89)	^†^86.2 (83.1–89.2)
Per-segment										
T2w-STIR			^†^95.2 (94.3–95.9)	^†^71 (65.6–75.9)	^†^29 (24.1–34.4)	^†^4.84 (4.16–5.67)	^†^96.8 (96.2–97.4)	^†^60.8 (55.9–65.5)	^†^0.83 (0.80–0.86)	^†^92.7 (91.9–93.8)
T2prep-SSFP			^†^92.6 (91.6–93.5)	^†^84.2 (79.8–88.4)	^†^15.8 (11.6–20.2)	^†^7.4 (6.6–8.4)	^†^98.2 (97.6–98.8)	^†^54.4 (50–58.7)	^†^0.88 (0.86–0.91)	^†^91.7 (90.6–92.7)
T2 map			96 (95.3–96.7)	87 (82.8–90.6)	13 (9.38–17.2)	4.02 (3.28–4.74)	98.5 (98.2–99.1)	69.6 (65.1–73.9)	0.92 (0.89–0.93)	95.1 (94.3–95.8)
T1 map			^†^94.5 (93.4–95.5)	^†^80.6 (75.9–85.2)	^†^19.4 (14.8–24.1)	^†^5.53 (4.51–6.61)	^†^97 (96.1–97.8)	68.9 (64.4–73.5)	^†^0.88 (0.85–0.90)	^†^92.7 (91.4–93.7)

Diagnostic confidence is defined according to the Likert scale; Confidence intervals are derived from bootstrapping with 1000 replicates

Only evaluable segments (384) and patients (65) are presented in this Table

*T2w*, T2-weighted; *T2prep*, T2 preparation; *T2 map*, T2 mapping; *T1 map*, T1 mapping; *STIR*, short-TI inversion recovery; *SSFP*, steady-state-free precession; *NPV*, negative predictive value; *PPV*, positive predictive value; *CI*, confidence intervals; *AUC*, area under the curve

*p < 0.05 in comparison with T2*w by mixed-effects ordered logistic regression. ^†^*p* < 0.05 in comparison with T2 map by the non-parametric Mann-Whitney *U* test

### Diagnostic accuracy of T2w-STIR, T2prep-SSFP, and T1/T2 maps for diagnosing IMH on evaluable segments and patients

On T2*w target slice, 19 of 50 evaluable STEMI patients (38%) showed IMH whereas none of the healthy controls was positive for IMH. Per-subject basis, T2 map, T1 map, and T2w-STIR showed the best overall diagnostic accuracy with very good or excellent sensitivity and specificity resulting in balanced negative and positive predictive values for IMH diagnosis (Table 2). In pairwise comparisons among the four CMR techniques, T2 map presented the highest diagnostic accuracy and AUC combined with the highest specificity and NPV (*p* < 0.05) (Table 2). Per-segment basis, 37 out of 384 evaluable segments (10%) had IMH based on T2*w. T2 map, T1 map, and T2w-STIR showed an excellent overall diagnostic accuracy combined with very good sensitivity and good specificity as well as high NPV for IMH diagnosis (Table 2). Similar to the per-patient analysis, T2 map combined the highest specificity, sensitivity, NPV, AUC, and diagnostic accuracy (*p* < 0.05) among all alternative techniques to T2*w.

### Quantitative target-slice data

Target-slice quantitative findings measured on T1 map, T2 map, and T2*w images are summarised in Table 3. In patients with IMH on T2*w, T2* relaxation time of IMH measured on multi-echo T2* imaging was significantly lower than the remote myocardium relaxation time (15.0 ± 3.1 vs 32.2 ± 6.5 ms, *p* < 0.001). T2 and T1 relaxation times of the oedematous myocardium were higher than those of the remote myocardium (*p* < 0.001), while T2 and T1 relaxation times of the hypocore were comparable with those of the remote tissue (*p* > 0.05). T2* relaxation time of IMH moderately correlated with hypocore T2 relaxation time (Spearman: 0.58, *p* = 0.014), whereas no correlation was found with hypocore T1 relaxation time (*p* > 0.05 both analyses). By general linear mixed model analysis, no evidence of difference in absolute hypocore size was found between T2*w and either T1 map (*p* = 0.854) or T2 map (*p* = 0.856) (Table 3). However, the relative hypocore size (% of slice) was larger on T2 map or T1 map than IMH as quantified on T2*w (*p* < 0.001 for both). Hypocore size measured on T2 map was comparable with that on T1 map (*p* = 0.669) (Table 3).

**Table 3:** Quantitative parameters of the target slice

Variable			
	T2*w	T2 map	T1 map
Oedema extent (g)	n.a	4.1 ± 2.0	3.0 ± 1.6
Oedema extent (% of slice)	n.a	31.6 ± 14.1	27.8 ± 14.4
Oedema relaxation time (ms)	n.a	61.7 ± 6.1	1219.8 ± 68.6
IMH or hypocore (g)	1.1 ± 0.9	1.4 ± 1.5	1.2 ± 1.0
IMH or hypocore (% of slice)	6.7 ± 5.1	9.1 ± 9.8*	10.8 ± 9.3*
IMH or hypocore relaxation time (ms)	15.0 ± 3.1	47.2 ± 4.8	1043.7 ± 74.2
Remote myocardium relaxation time (ms)	32.2 ± 6.5	45.4 ± 3.6	1007.4 ± 47.5
	LGE (infarct) / MVO		
Infarct size (g)	3.2 ± 2.4		
Infarct size (% of slice)	22.3 ± 13.5		
MVO extent (g)	1.6 ± 1.9		
MVO extent (% of slice)	9.2 ± 9.6		

*p < 0.05 in comparison with T2*w by general linear mixed model analysis. Only two outcomes (IMH size in grams and % of slice) have been formally tested among the three techniques. *IMH*, intramyocardial haemorrhage; *T2 map*, T2 mapping; *T1 map*, T1 mapping; *MVO*, microvascular obstruction; *LGE*, late gadolinium enhancement; *n.a*, not available

### Relationship between quantitative estimates of IMH on T2*w and hypocore on T2 map or T1 map

**Fig. 3:**
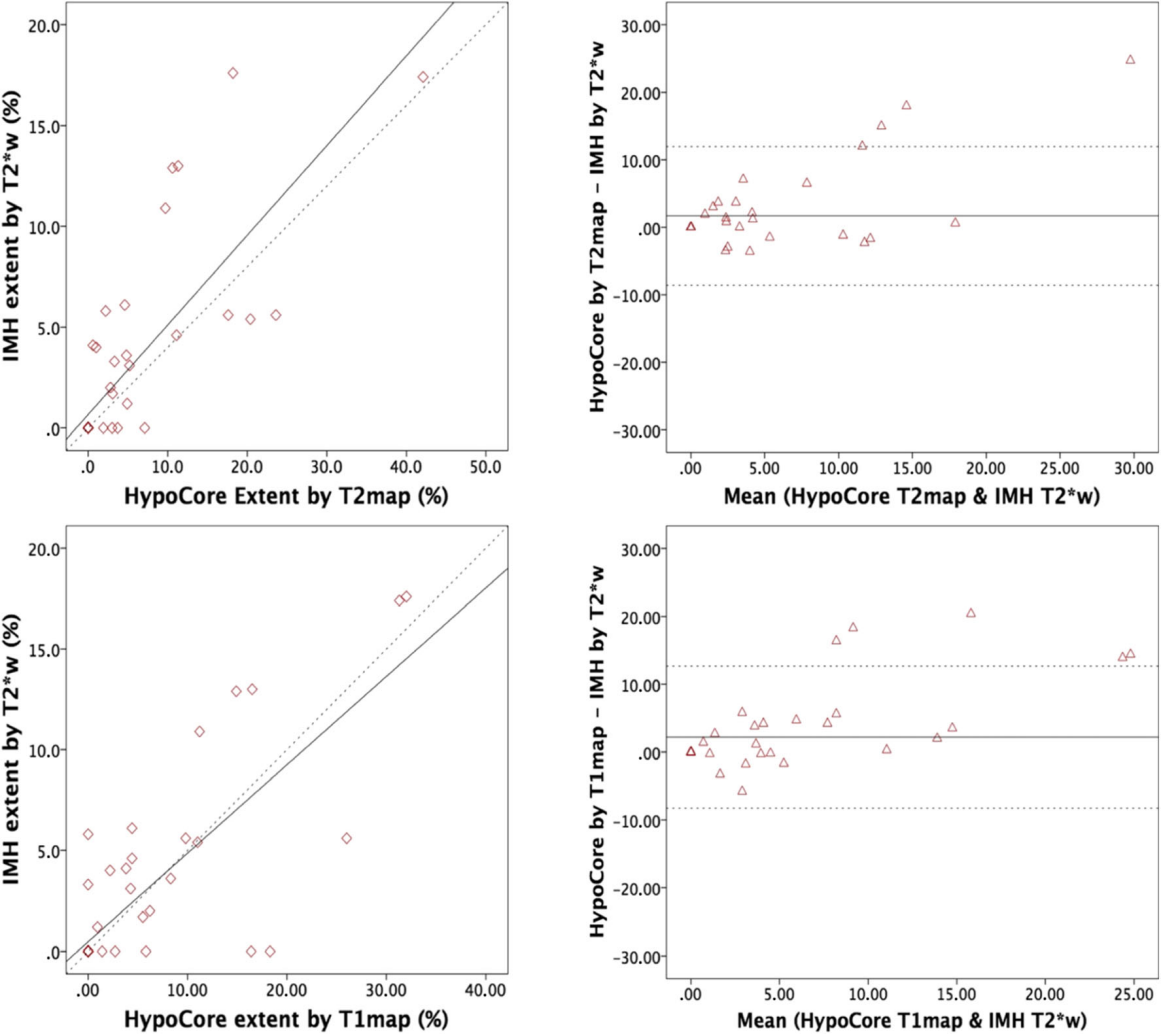
Correlations and Bland-Altman analyses between surrogate hypocores on T2 map, T1 map, and T2*w-based IMH. In panels **a** and **c**, scatter plots showing the correlation between the parameters. In Bland-Altman plots (panel **b** and **d**), the mean bias between the two CMR methods is portrayed by the solid horizontal line and the limits of agreement are displayed by the dotted horizontal lines. A solid line closer to 0 indicates better agreement between the CMR techniques. Correlation coefficients’ mean biases and limit of agreements are reported in Supplementary Table 4. *Abbreviations*: T2*w, T2*-weighted; T2 map, T2 mapping; T1 map, T1 mapping

There was a strong correlation between IMH extent on T2*w and hypocore size on T2 map (Spearman = 0.86, *p* < 0.001) with slight attenuation in the relationship with T1 map hypocore (Spearman = 0.73, *p* < 0.001) (Supplementary Table 4). On the Bland-Altman analysis, hypocore on T1 map (mean bias: 2.19, limits of agreement: − 8.27 to 12.65) and to a lesser degree on T2 map (mean bias = 1.69, limit of agreements: − 8.58 to 11.97) overestimated IMH gauged by T2*w but with an overall good agreement (Fig. 3) (Supplementary Table 4). Furthermore, ROC analysis showed an excellent and very good diagnostic accuracy for hypocore extent on T2 map and T1 map, respectively, in detecting IMH (AUC: 0.96 and 0.87, *p* < 0.001 for both). Hypocore extent of 1% and 0.5% on T2 map and T1 map had a sensitivity of 95%, 89%, and 93% and specificity of 87%, 83%, and 88% for IMH diagnosis, respectively**.**

### Reproducibility of the diverse techniques for IMH diagnosis and quantitative data for hypocore size

In the total population, T2 map showed the highest intra- and inter-observer reproducibility among comparators of T2*w for IMH diagnosis based on Cohen’s kappa statistic and intra-class correlation coefficients **(**Table 4**).**

**Table 4:** Intra- and inter-observer reproducibility of the diverse techniques for IMH diagnosis

Technique	Kappa statistic	*p* value	ICC single measure [95% CI]	*p* value	ICC average measures [95% CI]	*p* value
Intra-observer						
T2*w	0.92	< 0.001	0.92 [0.83–0.94]	< 0.001	0.96 [0.91–0.98]	< 0.001
T2w-STIR	0.67	< 0.001	0.6 [0.47–0.81]	< 0.001	0.80 [0.64–0.89]	< 0.001
T2prep-SSFP	0.78	< 0.001	0.79 [0.63–0.83]	< 0.001	0.88 [0.77–0.94]	< 0.001
T2 map	0.96	< 0.001	0.96 [0.92–0.98]	< 0.001	0.98 [0.96–0.99]	< 0.001
T1 map	0.91	<0.001	0.91 [0.84–0.95]	< 0.001	0.95 [0.91–0.97]	<0.001
Inter-observer						
T2*w	0.82	< 0.001	0.83 [0.65–0.92]	< 0.001	0.91 [0.79–0.95]	< 0.001
T2w-STIR	0.63	< 0.001	0.68 [0.40–0.84]	< 0.001	0.81 [0.57–0.91]	< 0.001
T2prep-SSFP	0.77	< 0.001	0.78 [0.50–0.91]	< 0.001	0.88 [0.67–0.95]	< 0.001
T2 map	0.86	< 0.001	0.86 [0.69–0.94]	< 0.001	0.93 [0.81–0.97]	< 0.001
T1 map	0.78	< 0.001	0.78 [0.57–0.89]	< 0.001	0.88 [0.73–0.94]	< 0.001

*ICC*, intra-class correlation coefficient; T2*w, T2*-weighted; *T2prep*, T2 preparation: *T2 map*, T2 mapping; *T1 map*, T1 mapping; *STIR*, short-TI inversion recovery; *SSFP*, steady-state-free precession; *NPV*, negative predictive value; *PPV*, positive predictive value; *CI*, confidence intervals

**Fig. 4:**
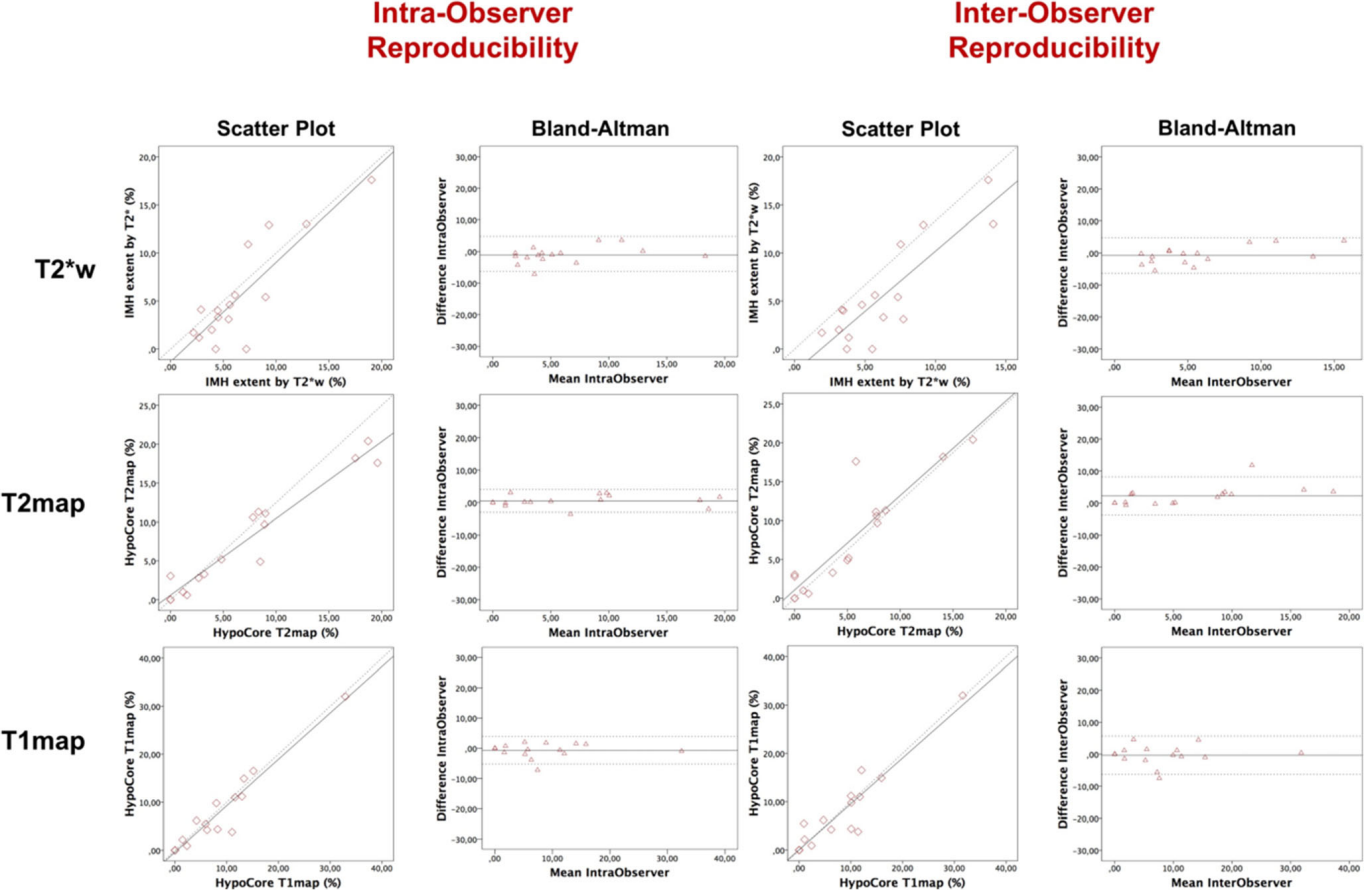
Intra- and inter-observer reproducibility of T2*w-based IMH and surrogate hypocore on T2/T1 maps. Pearson’s and Spearman’s correlation coefficients of the repeated measurements alongside relevant *p* values are displayed in Supplementary Table 5. In the Bland-Altman plots, the mean bias for repeated measurements of quantitative markers is portrayed by the solid horizontal line and the limits of agreement are displayed by the dotted horizontal lines. A solid line closer to 0 indicates better intra- and inter-observer variability. *Abbreviations*: T2*w, T2*-weighted; T2 map, T2 mapping; T1 map, T1 mapping

In 16 selected patients, intra- and inter-observer reproducibility for the quantification of IMH on T2*w and of the hypocore on T2 map and T1 map was good to excellent as detailed by very strong correlation coefficients. The Bland-Altman analysis indicated that T2 map had the best agreement in repeated measurements within the same observer (mean bias = 0.50) compared with T1 mapping (mean bias = − 0.66) and T2*w (mean bias = − 1.1) **(**Fig. 4) (Supplementary Table 5).

## Discussion

The major study findings can be summarised as follows. First, T2 map, T1 map, and T2w-STIR have a good-to-excellent per-subject and per-segment diagnostic accuracy for IMH diagnosis. The image quality, diagnostic confidence, and intra- and inter-observer reproducibility were higher for mapping techniques than T2w-STIR or T2prep-SSFP imaging. Second, hypocore on T2 map and T1 map correlated strongly but slightly overestimated the IMH extent with an overall good-to-excellent agreement. Third, hypocore extent quantified on T2 map and T1 maps showed better intra- and inter-observer reproducibility than IMH size measured on T2*w. Overall, these results strongly support the use of mapping for diagnosing and quantifying the hypocore as a surrogate measure of IMH as detected by multi-echo T2* imaging.

In our study, we used T2*w imaging as the clinical gold standard for IMH, which has been validated against histology and mass spectrometry for post-reperfusion haemorrhagic infarcts [10]. Although we paid particular attention in setting our clinical protocol by limiting the breath-hold duration and maximising B_*0*_ field homogeneity, as many as 8% of patients screened for study inclusion were finally excluded due to off-resonance artefacts on T2* imaging [19]. In the remainders, T2*w imaging performed well with respect to the overall image quality and diagnostic confidence in combination with a good intra- and inter-observer reproducibility for both qualitative (presence or absence of IMH) and quantitative data (IMH quantification). T2*w imaging, however, does not allow concomitant detection and quantification of infarct-related oedema, which could provide important additional information in STEMI in the early post-infarction phase [25, 26]. This limitation comes with the following drawbacks: Firstly, if T2*w imaging is used for IMH detection/quantification, an oedema-sensitive (e.g. T2 or T1 maps) technique has to be included in CMR protocol leading to prolonged scanning time and reduced patients’ comfort. Secondly, the proportion of patients excluded because of poor T2*w gives rise to a substantial increase in required study sample size [15–19]. Our results indicated T2 map, T1 map, and T2w-STIR had the best accuracy for assessing the presence or absence of IMH on per-subject and per-segment basis. However, T2w-STIR, alike T2prepSSFP, had lower image quality and diagnostic confidence compared with mapping techniques [27], and in 2 subjects (4%), T2w-STIR and T2prepSSFP images were excluded from the analysis because of very poor imaging quality. Within the remainders, the operator was uncertain or very uncertain in attributing or excluding IMH diagnosis based on T2w-STIR and T2prepSSFP images in 2 (3%) and 5 (8%) cases, respectively. In contrast, T2 map and T1 map had good-to-excellent image quality in combination with high or very high diagnostic confidence for IMH diagnosis in all cases. Our study results endorsed and expand previous knowledge about the use of mapping techniques for diagnosing and assessing IMH in STEMI patients. In particular, our findings are in line with those reported by Bulluck et al [28] in a smaller cohort of subjects and confirming the good sensitivity and specificity of T1 and T2 maps for IMH detection. That study was limited by the lack of a healthy control group (spectrum bias) and of direct comparison with T2wSTIR and T2prep-SSFP, which have been largely used in previous experimental and clinical studies for IMH detection [3–5, 7]. Our study superseded the limitation of the previous literature by reporting a properly chosen reference standard (i.e. T2*w), a head-to-head comparison of the most often used CMR techniques for infarct-related oedema and IMH imaging, and by including an age- and gender-matched healthy control group to reduce spectrum bias. We also detailed the precision of the diverse techniques for IMH quantification. Therefore, our study results provide a comprehensive background for researchers aiming to use CMR-based IMH identification and quantification for improving risk stratification in STEMI patients or for planning treatments to mitigate the deleterious I/R phenomenon.

It has to be acknowledged that T2/T1maps do not provide a direct measure of iron content within the infarct core. Based on our study results, and in particular the good diagnostic accuracy of T2/T1 maps in detecting IMH, it is reasonable to reserve T2* imaging to patients showing hypocore on T2 or T1 maps and withhold it in those without. In addition, given the settings of the current study which utilised a 1.5 magnetic field, dedicated studies on 3-T scanners are needed to test the diagnostic accuracy and precision of T2/T1 maps in the visualisation and quantification of IMH.

Finally, we found that hypocore by T2 map or T1 map slightly overestimated the size of IMH as quantified by T2*w imaging. Likewise, hypocore on parametric imaging and IMH represent two diverse aspects of I/R injury. In pre-clinical models of reperfused STEMI, histology data invariably showed a central necrotic core devoid of inflammation and blood flow due to the extensive irreversible microvascular damage alongside spotty areas of IMH [29–31]. It is likely that hypocore on parametric imaging represents the central necrotic core of reperfused infarcts, thus explaining why the hypocore areas slightly but consistently overestimate T2*w-based IMH. Furthermore, we found only a moderate positive relationship between the hypocore T2 values and T2* relaxation times and no correlation was found between T1 value and T2* relaxation time. This finding underpins that hypocore and IMH are two distinctive albeit closely interrelated phenomena of I/R injury. Concurrently larger hypocores on mappings are more likely to detect IMH as highlighted by the receiver operating curve analysis showing that a hypocore extent of 1% on T2 map had a sensitivity of 95% for diagnosing IMH. In IMH-positive cases, the T2 shortening is principally caused by magnetic susceptibility effects due to the compartmentalisation of paramagnetic deoxy- or methemoglobin inside the red cells, which usually occurs between 1 and 3 days after post-infarction IMH. In contrast, the determinants of T1 shortening in cases with IMH are likely more complex. Deoxyhaemoglobin, the predominant intra-erythrocyte form of degraded haemoglobin, is rather inaccessible to water molecules due its three-dimensional conformation resulting in negligible effect in T1 shortening. Methemoglobin, on the other hand, is prevalent in the haemorrhagic infarct between 4 and 14 days and exerts strong paramagnetic T1 shortening [8–12].

The study holds several limitations. Firstly, the head-to-head comparison of the diverse sequences was performed on one single slice (target slice). As a result, it was not possible to investigate whether the alternative techniques were able to visualise hypointense or hypo-T2/T1 regions within the infarcted myocardium not otherwise detected by T2* imaging. Histological validation was not possible in the current study and therefore, we were unable to investigate the contribution of diverse pathophysiological components of I/R injury on the relaxivity properties of the myocardium. Several studies adopted a T2* relaxation time < 20 ms as the gold standard for post-infarction IMH detection and quantification [5, 6, 9, 28, 31]. This cut-off is based on pathological values derived from explanted hearts of patients who died of hemochromatosis, and it has not been validated histologically in large animal models of I/R [32]. To the best of our knowledge, only Kali et al validated multi-echo T2* imaging with histology-based gold standard for post-infarction IMH [10]. Because IMH and microvascular obstruction (MVO) are closely related [29–31], it was not possible to discriminate the relative contribution of IMH or MVO to the relaxation time changes of the hypocore. Finally, T2 relaxation time of the hypocore region cannot be used to assess the severity of IMH.

In conclusion**,** in reperfused STEMI patients, the hypocore on T2 map or T1 map is an accurate and precise surrogate metric of IMH overcoming the limitations inherent to T2* imaging.

## Electronic supplementary material

ESM 1 (DOCX 200 kb)

## Abbreviations

CMR: Cardiovascular magnetic resonance
I/R: Ischemia/reperfusion
IMH: Intramyocardial haemorrhage
LGE: Late gadolinium enhancement
LV: Left ventricle
MVO: Microvascular obstruction
STEMI: ST-segment elevation myocardial infarction
T2*w: T2*weighted
T2prep-SSFP: T2-preparation steady-state-free precession
T2w-STIR: T2-weighted short-TI inversion recovery
