# Supplementary material for: Head-to-head comparison of multiple cardiovascular magnetic resonance techniques for the detection and quantification of intramyocardial haemorrhage in patients with ST-elevation myocardial infarction
Source: Eur Radiol. 2020 Sep 14;31(3):1245–56. doi: 10.1007/s00330-020-07254-1 (PMC7880961; doi:10.1007/s00330-020-07254-1)
Supplement: Supplementary file 1 — (DOCX 200 kb) [file 330_2020_7254_MOESM1_ESM.docx]

**SUPPLEMENTAL MATERIAL**

**Cardiovascular Magnetic Resonance Protocol – Target-Slice**

**Selection**

The target-slice was selected based to the stack of short-axis cine images acquired over the whole left ventricle. Cine images were promptly evaluated by an experienced operator (>10 years of experience in CMR). Based on wall motion abnormalities a base-to-apex direction of infarction can be determined (A), and the image showing the most extensive wall motion abnormalities located in the middle of infarct region was deemed as the *target-slice* (in red in the cartoon; B short-axis view). The most basal and the most apical slices in the infarct base-to-apex direction were excluded (dashed lines in the cartoon; A). The gray region in the cartoon depicts the myocardium with wall motion abnormalities (anterior STEMI); LA: left atrium; LV: left ventricle.


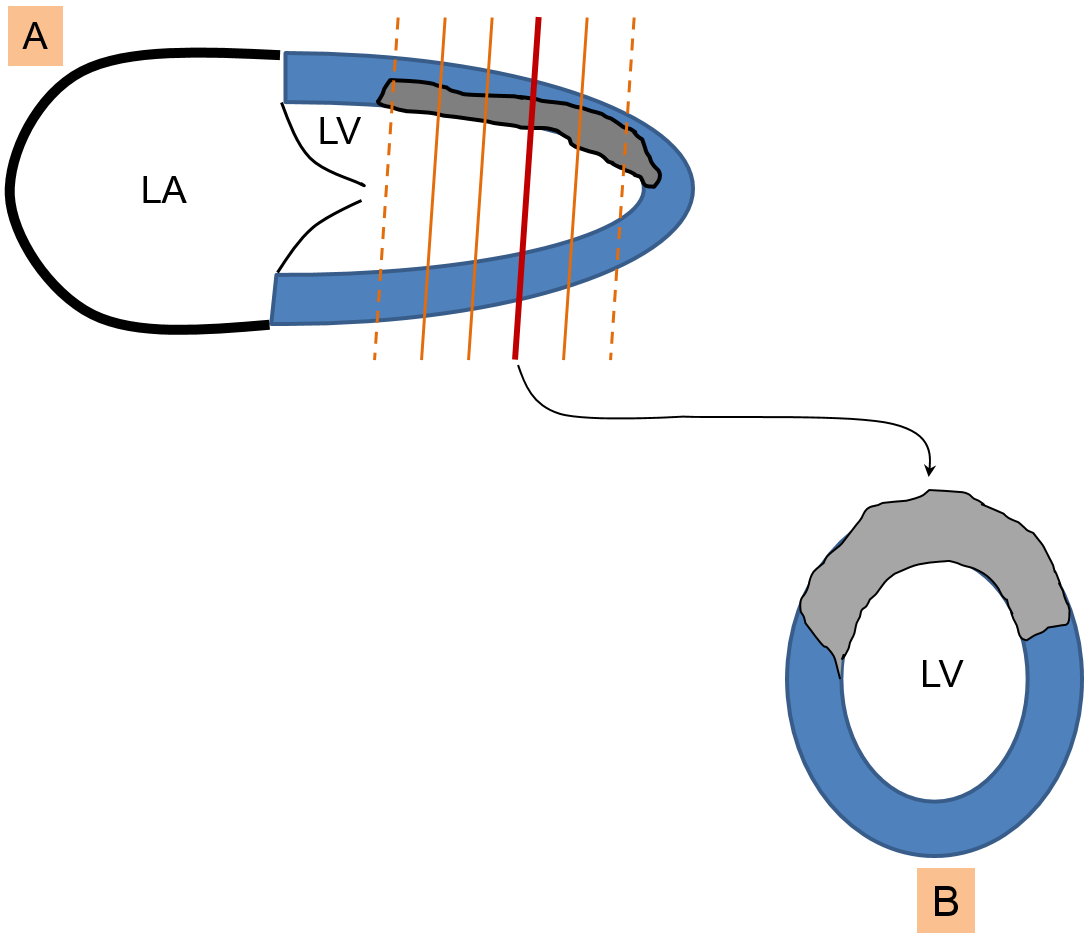


**Supplementary Table 1. CMR sequence parameters**

| **Technique** | **Sequence** | **Parameters** | **Notes on Image Generation** |
| --- | --- | --- | --- |
| Cine imaging | Breath-hold segmented steady state free precession (SSFP) | Flip angle:56°;  TR/TE:37.1/1.19 ms, BW: 925 Hz/Pixel; voxel size: 1.4x1.4x6.0mm; cardiac phases: 25 |  |
| T1map | 2D Breath-hold MOdified Look-Locker Inversion-Recovery (MOLLI) | Acquisition Sampling Scheme:  a) pre-contrast (native): 5(3)3  b) Post-contrast: 4(1)3(1)2  Read-out: single-shot SSFP with trigger delay in mid-diastole.  Flip angle: 35°; TR/TE: 272/1.2 ms; BW: 1085 Hz/Pixel; voxel size: 1.5x1.5x8mm^3^; PAT: 2; number of inversion pulse (180°) 2 and 3 for native and post-contrast, respectively | Generation of inline motion corrected pixel-based T1-maps by acquiring a series of images over several heartbeats with shifted T1 times corrected by RR duration |
| T2map | 2D Breath-hold T2-prepared SSFP sequence | Read-out: single-shot SSFP with trigger delay in mid-diastole.  Flip angle: 70°, TR/TE: 193/1.06 ms; BW: 1184; voxel size: 1.9x1.9x8mm, PAT: 2 | Generation of inline motion corrected pixel-based T2-maps by acquiring 3 images with different T2 preparation time (0, 25, 55 ms) with a gap of 2 RR interval within 1 breath-hold |
| Multi-echo T2* | Breath-hold Black Blood 2D spoiled gradient echo | Read-out: single-shot gradient echo with trigger delay in mid-diastole.  Flip angle: 70°, TR 700 ms; 8 echo time from 2.08 to 16.22 ms (∆TE: 2.00 ms); BW: 814; voxel size: 1.6x1.5x8mm, PAT: 2 | Generation of inline motion corrected pixel-based T2*maps by acquiring 8 images with different echo times with a gap of 2 RR interval within 1 breath-hold |
| T2STIR | Black-blood 2D short-TI turbo spin echo | Read-out: turbo spin echo with trigger delay in mid-diastole.  Flip angle: 180°, TR/TE: 750/55 ms; trigger pulse: 2 RR; TI: 180ms; BW: 849; voxel size: 1.4x1.4x8mm, PAT: 2 | . |
| T2prerSSFP | Bright blood 2D T2prep SSFP | Read-out: turbo spin echo with trigger delay in mid-diastole.  Flip angle: 90°, TR/TE: 184/1.3 ms; trigger pulse: 2 RR; T2prep: 60ms; BW: 651; voxel size: 1.3x1.3x8mm, PAT: 2 |  |
| Late  gadolinium enhancement | 2D breath-hold segmented T1-weighted gradient echo inversion recovery with phase sensitive reconstruction (PSIR) | Flip angle: 35°; TR/TE:848/3.8 ms; BW: 130 Hz/pixel; voxel size: 1.3x1.3x8.0 mm; PAT:2, number of segments: 20 to 30 |  |

TR: repetition time; TE: echo time; BW: bandwidth; PAT: parallel imaging acquisition technique

**Target-slice analyses**

**Qualitative analysis** consisted in visual assessment of ischemia-related edema and hypo-core in the target-slices. The hypocore was defined as hypo-intense / reduced relaxation time region embedded within hyperintense/increased relaxation time of the edematous infarcted myocardium. Microvascular obstruction on LGE was defined as hypointense core within hyperintense infarcted myocardium. The operators were freed to choose the most appropriate windowing setting for T2w-STIR, T2prep-SSFP and LGE, whereas T2/T1maps were interpreted on the reconstructed pixel-wise parametric images using the customary scale range of the vendor (0-200 ms for T2map; 0-2000 ms for T1map). When present, the operator allocated hypocore to a specific segment based on LV segmentation of American Heart Association [1].

**Quantitative analysis** was performed in T2/T1maps, T2*w and LGE target-slices images. Endocardial and epicardial contours were manually traced excluding blood-pool and epicardial adipose tissue. A region-of-interest (ROI) was traced in the mid-layer of non-infarcted remote myocardium devoid of artifacts. Infarct-related edema was quantified as myocardium >2 standard-deviations of the mean relaxation time of the remote myocardium. Pixels exceeding this cut-off but not belonging to the infarct vascular territory were excluded. Hypocore, if present, was quantified by manual tracing. Thus, hypocore corresponded to myocardium within the edematous tissue having a relaxation time ≤2 standard-deviations of the mean relaxation time of the remote myocardium. On LGE target-slice image, infarct-size was quantified as myocardium with a signal-intensity exceeding the mean signal-intensity of remote myocardium by >5 standard-deviations [2]. If present, MVO, was quantified by manual tracing. Accordingly, MVO corresponded to hypointense region embedded in the hyperenhanced myocardium (LGE positive) showing signal-intensity≤5 standard-deviation of the remote myocardium signal-intensity. Target-slice edema, hypocore, infarct-size and MVO were expressed as absolute (grams of tissue) or relative (% of target-slice) values.

**Left ventricle Image Analysis**

LV volumes, mass, and ejection-fraction were calculated by manually delineating endocardial and epicardial borders in the stack of short-axis cine images. The extent of infarct-related edema, hypocore, infarct-size and MVO were calculated for the entire LV using the same post-processing analysis as detailed above for the target-slice, and then summing up the results of each slice. The results of LV analysis are reported in the Table 2 (below).

**References**

1. Cerqueira MD, Weissman NJ, Dilsizian V, Jacobs AK, Kaul S, Laskey WK. Standardized myocardial segmentation and nomenclature for tomographic imaging of the heart. Circulation. 2002;105:539-542.
2. Symons R, Pontone G, Schwitter J, Francone M, Iglesias JF, Barison A, Zalewski J, de Luca L, Degrauwe S, Claus P, Guglielmo M, Nessler J, Carbone I, Ferro G, Durak M, Magistrelli P, Lo Presti A, Aquaro GD, Eeckhout E, Roguelov C, Andreini D, Vogt P, Guaricci AI, Mushtaq S, Lorenzoni V, Muller O, Desmet W, Agati L, Janssens S, Bogaert J, Masci PG. Long-Term Incremental Prognostic Value of Cardiovascular Magnetic Resonance After ST-Segment Elevation Myocardial Infarction: A Study of the Collaborative Registry on CMR in STEMI. JACC Cardiovasc Imaging. 2017 Aug 11. pii: S1936-878X(17)30620-4. doi: 10.1016/j.jcmg.2017.05.023.

**Supplementary Table 2. CMR results of 50 STEMI patients.**

| **Variable** | **Patients**  **(n=50)** |
| --- | --- |
| LV-EDVi (ml/m^2^) | 76.4±20.5 |
| LV-ESVi (ml/m^2^) | 37.0±15.3 |
| LV-EF (%) | 50.2±13.9 |
| LV-Mi (g/m^2^) | 64.1±13.2 |
| Edema (g) | 15±12 |
| Edema (% LV) | 9±8 |
| Infarct Size (g) | 12.4±9.2 |
| Infarct Size (% of LV) | 11.9±9.2 |
| MVO n, (%) | 20 (36%) |
| MVO extent (g) | 3.8 (2.2-7.4) |
| MVO extent (% of LV) | 3.4 (1.4-6.5) |

LV: left ventricle; EDVi: end-diastolic volume index; ESVi: end systolic volume index; EF: ejection-fraction; MVO: microvascular obstruction; Mi: mass index

**Supplementary Table 3. Characteristics of the healthy-controls**

| **Variables** | **Healthy-Controls**  **(n=15)** |
| --- | --- |
| Age, years | 57±17 |
| Male gender n, (%) | 10 (66%) |
| Family History of CAD n, (%) | 1 (6%) |
| Diabetes n, (%) | 0 (0) |
| Hypertension n, (%) | 8 (53%) |
| Hypercolesterolemia n, (%) | 7 (46%) |
| Smoking n, (%) | 7 (46%) |
| **CMR Data** |  |
| LV-EDVi (ml/m^2^) | 69.1±14.9 |
| LV-ESVi (ml/m^2^) | 28.6±11.2 |
| LV-EF (%) | 60.3±10.3 |
| LV-Mi (g/m^2^) | 59.9±12.7 |

CAD: coronary artery disease; CMR: cardiovascular magnetic resonance; LV: left ventricle; EDVi: end-diastolic volume index; ESVi: end-systolic volume index; EF: ejection-fraction; Mi: mass index.

**Supplementary Table 4. Comparison between T2*w-based IMH extent and hypocore size on T2map and T1map.**

|  | **T2map** | **T1map** |
| --- | --- | --- |
| **Correlation Coefficients** |  |  |
| *r* Pearson’s | 0.79* | 0.79* |
| Spearman’s Rho | 0.86* | 0.73* |
| **Bland-Altman Analysis** |  |  |
| Mean bias | 1.69 | 2.19 |
| Limits of Agreement | -8.6/12.0 | -8.3/12.7 |
| ∆ Limits of Agreement | 20.6 | 20.9 |

∆ Limits of agreement indicate the absolute range comprised between the negative and positive limit of agreement

*Indicates significant (P≤0.001) correlation between T2*w based IMH extent and either T1map or T2 map hypocore size

T2map: T2-mapping; T1map: T1-mapping;

|  | **T2*w** | **T2map** | **T1map** |
| --- | --- | --- | --- |
| **IntraObserver Correlation Coefficient** |  |  |  |
| Pearson’s | 0.86* | 0.96* | 0.96* |
| Spearman’s Rho | 0.75* | 0.92* | 0.92* |
| **InterObserver Correlation Coefficient** |  |  |  |
| Pearson’s | 0.86** | 0.91* | 0.93* |
| Spearman’s Rho | 0.69* | 0.91* | 0.86* |
| **Bland-Altman Analysis**  **IntraObserver** |  |  |  |
| Mean bias | -1.09 | 0.50 | -0.66 |
| Limits of Agreement | -6.34/4.15 | -3.03/4.03 | -5.23/3.94 |
| ∆ Limits of Agreement | 10.49 | 7.06 | 9.18 |
| Coefficient of Variation | 0.38 | 0.24 | 0.23 |
| **Bland-Altman Analysis**  **InterObserver** |  |  |  |
| Mean bias | -0.74 | 2.21 | -0.34 |
| Limits of Agreement | -6.29/4.81 | -3.76/8.18 | -6.35/5.67 |
| ∆ Limits of Agreement | 11.10 | 11.94 | 12.02 |
| Coefficient of Variation | 0.39 | 0.39 | 0.33 |

**Supplementary Table 5. Intra- and InterObserver variability for T2*w-based IMH and hypocore size on T2map and T1map**

*indicates very significant (P≤0.001) correlation coefficient among repeated measurements with the same technique and the same or different operator. **indicates significant (P<0.05) correlation coefficient among repeated measurements with the same technique and the same or different operator. ∆ Limits of agreement indicate the absolute range comprised between the negative and positive limit of agreement; *Indicates significant (P≤0.001) correlation between T2*w based IMH extent and either T1map or T2 map hypocore size;

T2map: T2-mapping; T1map: T1-mapping;
